# Insulin Resistance in Polycystic Ovarian Syndrome

**DOI:** 10.7759/cureus.30351

**Published:** 2022-10-16

**Authors:** Ananya Purwar, Shailesh Nagpure

**Affiliations:** 1 Pathology, Jawaharlal Nehru Medical College, Datta Meghe Institute of Medical Sciences, Wardha, IND; 2 Pharmacology, Jawaharlal Nehru Medical College, Datta Meghe Institute of Medical Sciences, Wardha, IND

**Keywords:** obesity, infertility, pathogenesis, syndrome, insulin, hormonal, resistance

## Abstract

Polycystic ovarian syndrome (PCOS) is one of the readily recognised endocrine gland illnesses in women, with an incidence range from 2.2% to 26% in India. Patients experiencing PCOS experience issues involving irregular menstrual periods, hirsutism, acne, being overweight, and impotence. Long-term, low-grade inflammation has emerged as a crucial factor leading to PCOS. A rise in glucose levels may stimulate oxidative stress and a troubling reaction from mononuclear cells (MNC) of females with PCOS, which normally do not rely on fat. This is required because MNC-derived macrophages are the major source of cytokine synthesis in big adipose tissue and similarly encourage adipocyte cytokine production. In summary, data reveal the substantial risks of insulin resistance in obese people who are suffering from PCOS. The findings of this specific lesson indicated that individuals with the conventional PCOS phenotype had obesity and higher insulin levels and insulin resistance, neglecting the absence of BMI differences from other phenotypes. These data show that insulin resistance is the most significant pathophysiological trait in people with PCOS.

## Introduction and background

Polycystic ovarian syndrome or PCOS is an endocrine condition which affects females at their reproductive age. Typical features include irregular menses, hyperandrogenism, and PCOS characteristics [1]. PCOS raises the risk of type 2 diabetes mellitus. Diseased women display resistance to insulin, independent of fat [2]. PCOS was assumed to occur from functional ovarian hyperandrogenism (FOH) generated by insufficient production of androgen [1]. PCOS uplifts major complications like diabetes mellitus in females. One in every five to six females encounters irregularity in their menses and major issues surrounding impotence. Tension, obesity, and differentiation in hormone levels are the primary cause globally [3]. The following are the four basic elements of the physiological foundation of PCOS: 1. Disorders of gonadotropin hormone synthesis. 2. The entry of insulin resistance. 3. The impact of the existing increased body fat [4]. The initiation of PCOS often develops throughout adolescence. It is characterised by greater alterations in different hormone levels. The premise is that the foundation of its diagnosis must depend on the existence of at least two of the following three criteria: hyperandrogenism, persistent anovulation, and polycystic ovaries. Many signs of PCOS include acne, irregular menses, and hyperinsulinemia. Youngsters who are diagnosed with PCOS are at a greater risk of acquiring health-related concerns in the near future, such as diabetes, cardiovascular disease, and impotence [5].

Several ideas have been provided to explain the aetiology of PCOS. The function of insulin resistance (IR) which is independent of fat is significant. It is associated to resulting in hyperinsulinemia, which drives excessive ovarian androgen production. Additionally, obesity-related inflammation may have relevant repercussions for ovarian composition [6]. Overweightness, insulin resistance, and hyperinsulinemia are the key metabolic anomalies influencing PCOS patients. Hyperandrogenism, infertility, and menstrual abnormalities are the most prevalent symptoms in females with PCOS. Hypertension, type 2 diabetes, and heart disease are frequent in these people [7]. These females have erratic gonadotropin absorption and androgen biosynthesis from the adrenal and ovaries, spurred by an elevated level of insulin. According to specialists, it is regarded that the fundamental feature of PCOS is hyperandrogenism. Various ideas suggest dissimilar features for understanding PCOS indices, such as a primary enzymatic default in adrenal and ovarian steroidogenesis. Damage in gonadotropin-releasing hormone excretion that indorses luteal hormone (LH) emission or alterations in insulin movements that produces insulin resistance are also some of the postulated features [7]. Most women with PCOS are metabolically resistant to insulin, either owing to genetic propensity or obesity. But some women with classic PCOS don't demonstrate insulin resistance [7]. Lately, epigenetic processes have been intricated in the pathophysiology of PCOS. This data implies that women with PCOS have a distinct epigenetic regulation, which might be triggered by a contradictory invitro environment or by post-delivery environmental variables such as lifestyle and or being overweight [8]. Overweightness is causative of an elevated cause of infertility. Overweight females have worse generative results regardless of the technique of conception, and a greater BMI is connected with inferior productiveness [9].

## Review

Relationship between PCOS and insulin resistance

 Insulin resistance is amplified by obesity. Inherent insulin resistance in PCOS is attributed to inappropriate reaction to insulin in metabolically active marginal tissues including adipose tissue and skeletal muscle [10]. Obese females with PCOS are more susceptible to insulin resistance, which might lead to abnormal glucose and lipid catabolism. Moreover, increasing insulin lowers the circulating amount of sex hormone-binding globulin (SHBG) and promotes free androgens, which constrains follicle formation resulting in irregular menses and impotency [11]. PCOS females had a significantly larger intake of a diet heavy in sugar (white bread and fried potatoes) [12]. Different adipokines are discharged from the fatty tissues, which have varying effects on insulin resistance. Some, such as visfatin, may stimulate the insulin receptor and have insulin-like activity, although adiponectin has an insulin-sensitizing effect. Adiponectin, released by the adipocyte, is a rich protein that occurs as multimers. It encompasses high, low, and middle molecular weights. Though studies showed the association between adiponectin and PCOS sovereign of BMI, others demonstrated that adiponectin levels were harmfully associated with BMI. These adipokines may be measured as markers of insulin resistance in PCOS patients regardless of BMI. The consumption of total fats, saturated fatty acids, and cholesterol should be reduced for easing the growth of diabetes and heart disorders. They impact the dysfunction of the ovaries [13]. Excessive insulin invention may activate insulin receptors of the pituitary gland to issue luteinizing hormone and worsen the excretion of androgen by the ovary and glands. It may restrict the formation of hepatic SHBG and boosts the levels of free testosterone. Excessive androgen excretion may lead to acne and alopecia signs and may impede the development of ovarian follicles [14].

Methods of detection of PCOS

The circular RNA (circRNA) chip is utilised to identify the difference in the expression of circRNA in the granulosa cells of PCOS patients [15]. Primary granulosa cells were obtained from patients with and without PCOS (controls, n = 32) during oocyte recovery. The girls with PCOS were arbitrarily assigned to undergo therapy. The reactive oxygen species level was resolute by spectrophotometry and fluorescence microscopy [16]. The androgens that are typically unrushed include total testosterone, free testosterone, calculated bioavailable testosterone, and calculated free testosterone (calculated FT) using the method of Vermeulen et al. [17]. If the prolactin levels were normal (equal to or less than 25 ng/ml), the patient was declared normal. If the prolactin levels were >25 ng/ml, estrogen stimulates prolactin production. Persistently high level of estrogen levels occur generally in women with PCOS and leads to mild prolactin elevation [18].

Insulin resistance biomarkers in PCOS patients

At the same time, there was a rise in levels of the other investigated agents (e.g., preptin, gremlin-1, neuregulin-4, xenopsin-related peptide, xenin-25, and galectin-3) [19], fully released by mature adipocytes. Decreased plasma concentration of adiponectin is connected with insulin resistance, obesity, type 2 diabetes mellitus, and cardiac problems. There is an evident elevation in leptin levels in obese PCOS patients compared to lean women with PCOS wheels, with a misleading connection between insulin sympathy and leptin levels in both PCOS groups [20]. Apelin is a peptide that is not accessible from bovine stomachs but is found in various other organs and also in visceral and subcutaneous tissues. It was revealed that apelin was increased in PCOS patients [21]. Lower kisspeptin serum levels in PCOS women than in controls were found. It is also found that there are significantly higher kisspeptin levels in normal-weight PCOS subjects than in obese women with the disease with a negative relation to BMI, androgens, fasting insulin levels, and Homeostatic Model Assessment for Insulin Resistance (HOMA-IR) [21]. Irisin levels were significantly higher in both groups of PCOS patients whether lean or obese but significantly higher in obese patients than in lean women [20].

Depression related to insulin resistance in PCOS

PCOS is of medical and societal health concern since it is relatively frequent, causing sickness in many females of the reproductive age group. It has important and varied therapeutic repercussions, including reproductive, metabolic (insulin resistance, impaired glucose tolerance, type 2 diabetes mellitus, and unfavourable cardiovascular risk profiles), and psychological aspects (amplified anxiousness, sorrow, and awful quality of life) (amplified nervousness, sadness, and lousy quality of life). Females with PCOS may present with various ranges of clinical symptoms, including mental issues (lower value of life, poor confidence, sadness, and anxiety) [22]. In females with PCOS, nervousness appears to differ in a pattern similar to that of hyperandrogenemia and insulin resistance, independent of age and BMI. The pathogenesis fundamental to the association of mental illnesses with androgen surplus and insulin resistance in PCOS remains to be clarified [23]. Studies reveal that females experiencing PCOS have greater mental problems than non-PCOS females. The frequency of anxiety in females with PCOS has been as high as 40% with mental problems like depression and eating disorders. Research has confirmed that being overweight and solitude are connected to the general population and that women with PCOS are typically fatter than non-PCOS females in general. It might be considered that obesity may promote emotional instability in females with PCOS. Moreover, it might be questioned that some extra elements of PCOS, such as infertility, hirsuteness, acne, and physical unpleasantness, could lead to emotional unwellness in females with PCOS. Overall, connected to enhanced inflammatory indicators, sadness is well documented to be associated with aberrant cortisol excretion. Females with PCOS exhibit inconsistent cortisol secretion despite mood changes [24].

Treatment and prevention

According to several research studies, many herbs may be utilised to recover various aspects of PCOS. Herbal medications restore generative disorders and perform a key function in harmonising hormonal balance and menses [25]. Hyperglycemia decreases hepatic synthesis of SHBG, which increases free androgens in the blood circulation, while insulin resistance promotes the creation of androgens by the theca cells. PCOS often develops in women with inadequate aliment and limited physical activity [26]. Obesity is not a component of the phenotypic in many places of the world. Obesity is likely not a determinant of PCOS, as there are equal chances of lean and thin women getting diagnosed with PCOS. Obesity deteriorates several phenotypic aspects, including cardiac illnesses such as glucose bigotry and dyslipidemia. It is also connected with a poor response to sterility therapy and increases pregnancy problems. Generally, most treatments for obesity, such as bariatric surgeries or weight-reducing surgeries, reach a large drop in weight, and advances in the PCOS phenotype, leading to weight loss in overweight females, remain one of the preceding therapies [27]. Health may be harmed by bad eating. Diet may have a vital effect in modifying gene expression, as well as it may potentially contribute to the cause of illnesses. Gynaecological ailments mainly concern the female venereal system and encompass benign and malignant cancers, infections such as COVID-19, and endocrine problems [28]. The function of aerophilic workouts, including continuing aerobic exercise training and high‐intensity interval training in PCOS organization, has been found. Research shows aerophilic exercises may benefit cardiometabolic and generative health in females with PCOS and those who are overweight [29]. Vigorous existence treatments must be incorporated into the organization plan for all teenagers with PCOS since a high percentage of them are overweight or are in danger of obtaining excessive weight. Lifestyle improvements encompass several elements, including nutritious eating, exercise, and reduced laziness. The interferences should also affect parental conduct. Dedication to lifestyle alterations may be strengthened by the treatment of mental variables like stress, body image worries, and disordered eating, which are frequent in the younger generations. Two comprehensive studies of lifestyle therapies in women with PCOS reveal improvements in weight. Lifestyle adjustments like management of weight, eating healthy food, and avoiding junk food have significantly reduced the chances or helped teenagers recover from PCOS [30].

Discussion

Many studies show the pretty joint influence of obesity and insulin resistance on the overall inflammatory response, indicating PCOS in a major way. It is expected that these females would mostly have elevated rates of obesity, diabetes, and cardiac risks, although there kind of has been no really clear difference between the mortality rates of regular versus women with PCOS. The requirement for long-term investigations is vital to essentially determine which phenotypes will exhibit extra well-being concerns at older age and whether there is a difference in morbidity rates among PCOS patients [31]. PCOS is becoming a generally more widespread condition among women of reproductive age with lifetime issues. One of the most actually exciting elements of this disease generally is its unclear very analytic criteria and great difficulty of characteristics, or so it was thought. In the meanwhile, additional studies in the genetics and pathophysiology of PCOS for the most part are required to regulate preventative risk factors and effective therapeutic methods for this illness [32]. PCOS basically is not only a multiplicative disorder but also a global condition, and its differential diagnosis is yet not totally understood, or so it was thought. Recently, the attitude of clinical practice has been a basically liberal adjustment and progress towards anticipation and the conventional treatments for disorders in a major way. Therapeutic techniques definitely are provided by hormonal contraceptives, antiandrogen medications, metformin, and inositols [33], which is quite significant. All patients should be well informed about the physical workouts and exercises which are needed to maintain a healthy lifestyle. Youths and obese females should definitely be inquired about for all intents and purposes physical work and advised promptly. A vigorous aerophilic exercise at least three times a week for 30 minutes or more for all intents and purposes is advised, which is fairly significant. A specifically heart rate monitor or the amount (volume) of oxygen the body uses while exercising as hard as one can (VO2max)-guided intensity levels (≥60% VO2max) are generally indicated to get insulin-related effects, which for the most part is quite significant. Yoga may be a helpful practice to integrate as a regular workout. Studies are done to validate its benefits for androgens and insulin response [34], which for all intents and purposes is fairly significant. Some lifestyle adjustments are known to help the symptoms and mental well-being of PCOS patients. This research is targeted at lifestyle improvement of PCOS patients by normalising insulin resistance, enhancing androgen status, and helping weight management, consequently promoting the avoidance of other linked noncommunicable illnesses. Millions of rupees may literally be saved if the country concentrates on alternative medications without sacrificing the health of the patients in any way.

## Conclusions

The relationship between PCOS and insulin resistance has proven that insulin particularly is a crucial hormone in females of the reproductive age group. Also, insulin activity in the central nervous system is necessary for ovulation. Research has shown that insulin resistance can lead to the production of smaller eggs or delayed production of eggs. Also, androgens have a substantial influence on insulin sensitivity and secretion. This idea translated directly into a new therapy for PCOS with insulin-sensitizing drugs (ISDs) in a subtle way. In addition, the possibility that androgen exposure during development may contribute to PCOS has received considerable attention since the very prenatal administration of androgens to non-human primates, sheep, and rodents can produce remarkable phenocopies of the syndrome. PCOS is a significant metabolic and reproductive illness linked with an elevated risk of diabetes mellitus throughout a lifetime in women suffering from it. Affected women literally have a specific disturbance in insulin action as a consequence of fairly lower insulin receptor activation, possibly mediated by receptor serine hyperphosphorylation and insulin receptor substrate 1 (IRS-1), or so it has been thought. This phosphorylation is produced by increased intracellular serine kinase activity. Insulin resistance in PCOS essentially is selective.
